# Supplementary material for: Are carbon emissions trading and green financial instruments synergistic? -Comprehensive quantitative research based on content analysis
Source: PLoS One. 2024 Mar 7;19(3):e0298601. doi: 10.1371/journal.pone.0298601 (PMC10919601; doi:10.1371/journal.pone.0298601)
Supplement: S1 Data — (ZIP) [file pone.0298601.s001.zip › Data/地级市Z_F工作报告.zip!/╔╜╬≈/┤≤═1⁄4╩╨/└┤╫╘╣1⁄2╓┌║┼í╛┬φ┐╦╩2╛▌═°í┐_2010.docx]

各位代表：

现在，我代表市人民政府向大会作工作报告，请予审议，并请市政协委员和其他列席人员提出意见。

一、2009年工作回顾

2009年，面对国际金融危机挑战和煤炭企业兼并重组阵痛的双重考验，我们紧紧依靠全市人民，在危机中抢抓机遇，在困难中寻求突破，在挑战中把握主动，着力推进大同经济社会发展的新跨越。

—— 经济发展在艰难中保持平稳，固定资产投资增幅较大。全市地区生产总值完成596.1亿元，增长2.3%；社会消费品零售总额264.7亿元，增长14.5%；财政总收入120.9亿元，下降1.1%；一般预算收入47.6亿元，增长2.0%；城镇居民人均可支配收入14585元，增长6%；农民人均纯收入3589元，增长7%。值得关注的是固定资产投资完成475.2亿元，增幅高达71.8%，为长远发展积累了基础。

—— 煤矿兼并重组取得阶段性成果，煤炭支柱地位进一步巩固。原有270座矿井政策性关闭为127座，最后兼并整合为64座，同煤、山煤、煤运、中煤等主体企业全部到位，总产能为4600万吨。矿井数量减少了206个，办矿主体减少了240个，产量增长了近一倍。煤炭企业从“多、小、散、乱”的困扰中走出来，朝着规模化、集约化、机械化、信息化的目标迈进。同煤塔山、同忻、东周窑三个千万吨以上现代化矿井，成为大同煤都的新地标，马道头千万吨矿井前期工作就绪。

—— 园区建设和招商引资起步，转型发展初见成效。中国重汽大齿公司投资12亿元、年产值30亿元的项目在装备制造园区开工建设。冀东水泥投资12亿元、年产500万吨水泥生产线一期工程竣工投产。蓝星集团在龙泉园区投资12亿元、年产3万吨氯丁橡胶项目投入试运行。国药集团与威奇达合作投资12亿元的原料药基地项目已完成土建基础。中海油与同煤合作投资300亿元开发40亿立方煤制天然气、华润投资建设30万吨啤酒生产线、上海复星与同钢合作年产300万吨钢、北方交通煤机制造、振东药业黄芪中药系列开发、庞大汽贸园区、香港中华煤气油页岩开发等项目积极推进。

—— 城市建设继续强力推进，为“三年大变样”目标实现奠定基础。投资46.7亿元新建改造城市道路38条、桥梁28座、总长105.48公里。拆迁各类违规、不协调建筑达268万平方米。新增城市绿化面积701万平方米。新铺设城市供水管网87公里，新铺设城市天然气管网113公里。新增集中供热面积840万平方米，拆除191家296台燃煤锅炉，全年二级以上天气341天，比上年增加34天，综合污染指数下降35.2%，降幅位列全省第一。垃圾发电厂和东郊污水厂中水回用工程投入运营。城市数字化管理平台启动，机械化清扫面积达到60%以上，城市规划展示馆对外开放。实施9个城中村改造，建筑面积209.9万平方米。

—— 文化复兴工程全面推进，“一主两翼”框架基本形成。东城墙、华严寺、善化寺、文庙、关帝庙、帝君庙、清真寺、纯阳宫、法华寺、华严寺广场等名城保护十大工程已完成90%的工作量，中国传统建筑神韵初步展现。云冈大景区建设整治工程取得实质性进展，景区周边一镇五村搬迁、339省道改线完成，景区内山堂水殿、游客中心、博物馆、商业街、研究院、演艺中心等景观、功能建筑主体基本完工。恒山大景区综合整治效果显现，历史街区、悬空寺景区、岳门湾建设进展顺利，吕律神祠修复竣工。

—— 基础设施建设实现突破，扩大内需机遇落地大同。争取国家和省资金28.09亿元，总量超过前二十年总和。荣乌、大呼、大天、大浑、浑广5条高速公路全部奠基开工，总长393公里，总投资202.6亿元，明年大同将实现高速公路由县县零公里到县县通的历史性跨越。总投资12.3亿元的208改线工程准备工作就绪。投资3亿元的机场改扩建工程列入国家扩大内需项目，已完成跑道改造工程。大西铁路客运专线开工建设，大张铁路客运专线前期工作顺利进行。

—— “三农”基础建设加强，农业产业化水平提升。龙头企业发展到124户，雨润集团百万头生猪屠宰项目开工建设。完成标准化养殖小区107个，新增温室大棚11260栋，新增水地5.2万亩。孤山水库、文瀛湖水库、万家寨引黄北干线等工程开工建设，万泉河引水工程竣工，大同现代农业示范区建设正式启动。“五个全覆盖”全面推进。198个校舍安全项目全部启动；村通水泥路完成790公里；安全饮水解决23万人；县乡医疗卫生机构达标率83%，村卫生室覆盖率100%；村通广播电视卫星接收工程完成1003个。

—— 民生工程投入巨大，社会事业协调推进。开工建设面积546.34万平方米、56055套安置房，已竣工84.4万平方米、10120套。大同棚户区改造的经验得到中央肯定，国务院在大同召开了全国城市和国有工矿棚户区改造现场会议。一中新校、二中初中部、实验中学初中部工程基本完工。市一医院、五医院、中医院、六医院、二医院放疗室、卫校等开工建设，三医院综合住院楼、医疗废物处理中心完工。发放救助资金5.6亿元。争取上级社会保障、就业补助资金6.99亿元。财政拨付费用13.94亿元，专项解决122户关闭破产企业36670名职工安置和善后问题。开展社会治安综合整治专项活动，强化安全生产管理，煤矿安全生产实现零死亡。

—— 制度建设不断加强，政府办事效率进一步提高。深化行政审批制度改革，将市级原有的411项行政审批事项精简压缩到182项，审批法定时限缩短二分之一。有效整合资源，理顺管理体制，先后完成商校、会校，一、二卫校，一、二技校，一、二物资，二医院、肿瘤医院，热力公司、供热公司等31家大单位、公司的合并重组，实现了资金、资源、人才利用的最大化。进一步转变政府职能，有序推进政府机构改革。严格各项制度，从源头上预防腐败，进一步规范工程招投标制度、土地出让制度、人事管理制度。坚持土地拍卖、挂牌出让，阳光作业，公平交易。违法建设得到有效查处和控制，建筑市场秩序进一步规范。行政事业单位坚持凡进必考，严把进人关，优选高素质专业人才。自觉主动接受人民代表大会及其常委会的法律监督，接受人民政协的民主监督。全年共办理人大代表建议141件和政协委员提案345件，办复率100%。

各位代表，过去的一年，成绩来之不易，奋斗充满艰辛。这些成绩的取得，是省委、省政府和市委正确领导，市人大、市政协监督支持的结果，是全市各级干部群众奋力拼搏的结果，也是各民主党派、无党派人士、工商联、人民团体、驻同人民解放军、武警官兵、公安干警和全市社会各界共同努力的结果。在此，我代表市人民政府，向所有关心支持大同改革、建设、发展的同志们、朋友们，表示衷心的感谢和崇高的敬意！

总结过去的一年，我们也清醒地看到存在的矛盾和问题，主要是“五个滞后”。结构调整滞后，产业发展单一的矛盾依然突出；对外开放滞后，招商引资落地项目少，内生动力不足的矛盾依然突出；民生改善滞后，拆迁与安置的矛盾依然突出；城市管理滞后，重建轻管的矛盾依然突出；队伍建设滞后，专业化人才短缺的矛盾依然突出。对于这些问题，我们一定要高度重视，认真加以解决。

二、2010年工作总体目标和任务

2010年是大同“一年一个样、三年大变样”的实现之年，也是五年基本实现文化名城、旅游名都、生态名邑、经济强市大目标的关键之年。历史经验表明，全面振兴大同，必须充分认识和发挥大同文化资源厚重、矿产资源丰富、土地资源广阔三大独特优势，全力打造大同的核心竞争力。这就要求我们必须站在更高的历史起点上，“登高壮观天地间”，以战略导向取代机会导向，以长远规划取代短期规划，以更大的胸怀、更大的气魄、更大的手笔，着眼长远谋划当前，站在明天建设今天，面向未来寻找支点，引领大同创造未来，赢取未来，走向未来。在前进的道路上，我们可能遇到这样那样的困难和干扰，但任何困难在坚持面前都是渺小的，任何干扰在坚强面前都是软弱的，任何艰难险阻都挡不住大同复兴的伟大历史进程。

做好今年的政府工作，要以邓小平理论和“三个代表”重要思想为指导，深入贯彻落实科学发展观，加快推进文化名城、旅游名都、生态名邑、经济强市“三名一强”建设步伐，继续坚持大规划引领，实施大投资方略，强化大项目支撑，打造大开放优势，抢占大发展高地，努力夺取经济社会发展的新胜利，为转型发展、绿色崛起，强势启动“十二五”规划奠定坚实的基础。

今年经济社会发展的主要预期目标是:地区生产总值增长8%，财政总收入增长7.5%，一般预算收入增长7%，工业增加值增长10%，全社会固定资产投资增长35%，社会消费品零售总额增长15%，外贸进出口总额增长5%，城镇居民人均可支配收入增长10%，农民人均纯收入增长10%，人口自然增长率控制在6‰以内，粮食总产量稳定在70万吨左右。主要约束性指标是:万元地区生产总值综合能耗下降6%，二氧化硫排放量控制在13.8万吨以下，化学需氧量排放量控制在4.68万吨以下，居民消费价格涨幅控制在3%左右，城镇登记失业率控制在4.2%以内。

实现以上目标，要重点做好以下工作。

（一）突出双城格局，打造中国特色城市大未来

城市是我们的家园，是经济发展的巨大引擎。城市化与工业化是现代化的两个轮子，加快推进城市化步伐，建设特色化的古今双城，是大同迈向现代化的重大战略。在城市化进程中，大同不被平庸的浪潮所淹没，不为时代的进步所抛弃，最根本的在于创造自己的特色。有特色的城市，才拥有生命，拥有未来，拥有高贵的价值。大同特色的灵魂在于一轴双城、古今兼顾的独特布局。穿越历史尘埃的古城巍然特立，给我们自信的底气；彰显时代水平的新区脱颖而出，给我们创造的豪气。在相当长的时期，我们忽略了自己城市独特的价值，持续不断的建设性破坏使特色沦丧，价值贬低，个性走向一般，高贵走向平庸。建设特色大同，是当代大同人沉重的历史责任。尽管特色化的道路充满风险和艰辛，需要付出巨大的成本，甚至生命的代价。但历史选择了我们，时代呼唤着我们，人民期盼着我们，我们别无选择，只能披肝沥胆，奋勇前行。一个最具中国特色的城市，是大同的未来和希望。

要加快旧城改造步伐，今年基本完成旧城区道路改造的任务。旧城区新建改造云中路、开源街、西环北延等28条、35公里道路，逐步加大旧城小街小巷整治改造的力度，实现旧城区道路网络体系标准化、整洁化、优质化，追求城市每一个细部的完美，从根本上改变人民群众的生活、出行条件。矿区实施同泉路到塔山工业园、206改造等3条14公里道路，整体提升城市基础水平。

要加快推进历史文化名城复兴工程。继续完善华严寺、善化寺、文庙、关帝庙、法华寺、清真寺、纯阳宫、帝君庙、东城墙、华严寺广场十项工程，尽快形成古城文化气场和氛围，让人民群众看到古城的希望和明天。启动南城墙、代王府、书院、府衙、城隍庙、北魏明堂、鼓楼东西街、钟楼南街步行街、清真寺广场、东小城十大工程，为争取在本届政府任期内基本完成古城修复的历史重任奠定基础。

要加快御东新区建设的步伐。开工建设御河东路南延、太和路等6条、40公里道路，基本形成新区道路框架。完成得大高速公路8公里改线，拓展御东核心区文瀛湖周边的开发空间。完成208一级路改建56公里，为御东新区发展创造基本条件。完成平城、兴云、北都、南环4座大桥，加强新区与古城的空间联系。开工建设博物馆、大剧院、图书馆、美术馆、体育中心等文化体育设施项目，完成政务大厅、一医院、五医院、中医院、卫校、十六中、实验小学等教育卫生项目。让御东新区成为城市兴奋的新亮点。

要充分发挥规划的龙头作用，提高县区城乡规划水平，以高水平的规划指导、控制未来。要维护规划的权威性、原则性和严肃性，坚决制止和打击违法建设行为，在规划指导下有序推进城乡建设。要持续加强和改进城市管理，实现清洁城市的最基本目标。充分利用城市数字化管理平台，全天候、全方位进行监管，建立城市管理的长效机制。加大环卫基础设施建设投入，大力度推行清扫机械化、收集容器化、清运密闭化、保洁全天化。坚持权力下放、重心下移，强化区、街道办、居委会城市管理的职能。提高全民环卫意识，培养全社会公共卫生习惯，推行“全民义务卫生活动日”，下大力治理垃圾围城的顽症，形成环境卫生齐抓共管的社会合力。从严惩处破坏城市公共设施、干扰城市秩序、损害公共卫生环境的各种不良行为。

要坚持以城带乡、城乡统筹，加快城乡基础设施建设。完成大呼高速公路建设，加快荣乌、大天、大浑、浑广高速公路建设进度，全力打造全市高速公路一小时经济圈。力争建设600公里“村村通”水泥（油）路。推进大西高速铁路建设，力争大张高速铁路尽快开工建设。全面完成机场飞行区扩建工程，开工新航站楼及其配套工程。

质量是建设的生命。百年大计，质量第一。必须从严优选建筑施工队伍，强化全过程、全方位的施工监理，严格业主责任制，决不放过任何一个环节的质量问题，从严从重奖优罚劣，确保政府工程经得起历史和人民的检验。要认真整顿建筑市场，健全质量监控体系，严把建筑材料关，提高混凝土搅拌准入门槛，依法打击假冒伪劣产品。强化全社会质量意识，建立健康、理性、负责的质量管理文化。

（二）突出文化复兴，打造世界旅游名都大品牌

大同以历史文化资源厚重闻名于世。北魏京华、辽金陪都、明清重镇是她三个最辉煌的时期，世界历史文化遗产云冈石窟、中国历史文化名城、国家风景名胜区恒山是她三张最闪亮的名片。依托丰厚的文化资源，发展文化旅游大产业，造福一方百姓，是国内外许多城市的成功经验。“他山之石，可以攻玉”。大同完全有条件也有可能成为世界旅游名都，成为国际旅游终极目的地。文化可以兴大同、名大同、富大同。文化是一个民族的精神高度。文化的力量是真正改变大同命运的决定性力量。大同社会每一次跃进，大同文明每一次升华，无不镌刻着文化进步的烙印。大同文化旅游产业要有明确的战略定位，有大战略、大胸怀，方有大视野、大世界。要立足北京，叫响全国，走向世界，融入大京都文化旅游圈。率先从京津突破，抢占制高点，顺势而为，乘势而上，让大同走向全国，进军世界。

要加快文化旅游大景区、大产业建设，打造“民族魂、中国牌、世界宝”的品牌效应。中国历史文化名城要做中国第一的大品牌，云冈石窟要做中国最好的大景观，悬空寺要做中国最奇的名胜区。今年七月底，云冈石窟所有园林、景观、服务、演艺、展览等工程要全面竣工，华严寺、善化寺、文庙、关帝庙、纯阳宫、法华寺等要具备对外开放条件。要推进恒山风景区、浑源历史街区、神溪湿地建设，逐步建设以古城堡、长城为核心的边塞文化景观，拓展和丰富大同文化旅游的内涵和空间。全力推动大同文化旅游向产业化、高端化、精品化、公益化方向发展。旅游产业载体，作为城市的文化支点，以历史的力量为城市加固，以艺术的经典使城市夺目，以支点的伟大使城市动容。

文化旅游产业的影响力、凝聚力、生命力，不仅要靠文化硬实力，还要靠文化软实力。以自觉的文化追求为目标，超越功利、世俗的纷扰，回到我们自身文化的源头，寻找我们的精神家园和独特优势。要高度重视发掘、整理、提升耍孩儿、罗罗腔、阳高二人台等非物质文化遗产，充分发挥大同文化名人的市场效应，打造具有浓郁地方特色、中外游客喜闻乐见的优秀剧目，形成品牌带动品牌的良性互动。要抓住京大高速铁路建设将要带来的一小时到达北京的空间优势，吸引北京和全国各地的雕塑家、画家到大同建立艺术创作基地，打造文化艺术之都。要精心营造古城浓郁的传统文化氛围，恢复传统的老字号、小吃街、手工作坊，形成中国传统文化展示的活标本。要高水平办好云冈文化艺术节和观音圣诞节、浴佛节、盂兰盆节、腊八节、祈年节等重大节庆、佛事活动，加大文化大同的营销力度。进一步加强与中国民间艺术家协会合作，建设中国民间文化艺术基地和中国民间艺术博物馆，举办中国民间文化艺术狂欢节、中国民间灯会、中华武功、民间文化艺术产品博览会等重大活动，让中国民间艺术节长期扎根大同，不断孕育、发展、传承、壮大，形成异彩纷呈、蔚为壮观的文化场景和文化大特色，成为大同旅游名都的文化品格、城市品位和时代品质。

（三）突出园区招商，打造经济强市硬实力

加快工业园区建设，做到空间集中开发、资源集约利用、产业集群发展、服务集聚配套，是发展低碳经济、实施经济强市战略的必由之路，也是城市发展战略规划提升、空间布局优化、招商引资落地的关键所在。工业由散点布局向集中布局、由混合布局向分区布局、由城内布局向城外布局，是工业化与城市化协调发展的大趋势。建设经济强市，必须把高标准园区建设摆在战略首位，重点抓好五大园区建设。

—— 装备制造园区。规划面积20平方公里，先期启动10平方公里，今年达到水、电、气、暖、道路、通信“六配套”，形成生产要素集聚、服务平台共享、开发政策优惠、办事效率快捷的品牌效应，强化园区招商的吸引力、号召力。对第一个入区项目—— 中国重汽大齿变速箱基地建设，要精心组织落实服务措施，确保年底实现大齿顺利搬迁、产能提升的目标。行动胜于任何美丽的言辞。良好的服务和效率，就是园区品牌建设的通行证，招商引资的金字招牌。

—— 医药工业园区。大同具有发展医药工业得天独厚的自然条件，医药产业已形成一定规模。现在的问题是空间布局不合理、产业要素不匹配，发展能量和潜力没有释放出来。大同医药应当有一个更大、更快、更好的发展。新规划的医药园区布局在距御东新区20公里的落阵营，规划面积20平方公里，首期启动6平方公里。今年要实现水、电、气、道路、通信“五配套”，启动医药企业搬迁工程。明年完成供热、污水处理配套，完善园区发展平台。要强化医药园区招商引资的力度，积极支持威奇达、仟源、普德等龙头企业做大做强，向销售突破100亿元冲刺。

—— 塔山循环经济园区。塔山园区目前已形成塔山、同忻两个千万吨、云峰300万吨的现代化矿井，总产能3300万吨，成为大同煤炭工业的标志。发展的基本模式是煤电一体化，粉煤灰、矸石综合利用和高岭土加工，形成循环产业链，发展能源低碳经济。今年要完善塔山园区路网建设，加快国投2×13.5万千瓦、同煤 2×30万千瓦热电联供等项目建设，加大招商引资，实施龙头项目，引领园区品牌。

—— 龙泉化工园区。继续强化与中国蓝星集团的合作，抓住杜邦公司产业升级的机遇，科学承接产业转移，尽快实现氯丁橡胶年产10万吨、世界最大的目标。瞄准TDA等重大化工项目，让蓝星集团在园区做大做强。

—— 左云煤制天然气园区。园区布局同煤东周窑、马道头、潘家窑三个千万吨矿井，以此为基础，推动与中海油的战略合作，建设40亿立方煤制天然气项目，实现大同煤炭由输煤、输电到输气并举的战略转变。左云园区要加快规划设计、规划环评、水资源论证、项目选址、土地征用等前期工作，积极为煤制天然气落地创造条件。

以园区为载体发展优势产业，必须借助资本市场强大的融资功能，为企业做大做强注入活力。支持同煤集团、大秦铁路公司实现整体上市，推进仟源药业、普德药业、新成特炭、华晟果蔬等企业加快股改上市，让更多的企业插上腾飞的翅膀。要大力培育和扶植民营经济在园区发展，落实激励政策，创造宽松环境，推动民营经济不断发展壮大。

电力产业是大同的传统优势产业，要积极推进二电厂2×100万千瓦、同煤与京能2×100万千瓦两个特大型电厂的前期工作，力争明年开工建设，向装机容量1000万千瓦目标进军。煤炭兼并重组是大同煤炭产业重大变革，必须抓紧推进技改扩产等基础性工程，缩短改革的阵痛，推动地方煤炭产业健康持续发展。

（四）突出绿色崛起，打造生态名邑新名片

坚持以科学发展观为指导，树立生态文明理念，建设宜居、利居、乐居城市，实现生态文明与社会文明协调统一、人与自然和谐相处的环境友好型社会，是大同“三名一强”战略目标之一。大同气候干旱，风沙较大，缺水严重，生态环境脆弱，必须加大生态文明建设的力度，坚持“生态立市、绿色崛起”的战略不动摇，把绿化大同作为基本政策，长期坚持下去。今年要启动国家园林城市申报工程，力争三年内实现这个目标。

要继续做好城市生态建设工作，实现“水”与“绿”的完美结合、“人”与“城”的和谐相处。今年重点抓好12项园林绿化景观工程。一是文瀛湖景观工程。年底完成文瀛湖防渗蓄水工程、湖西岸绿化景观工程，明年全部竣工，让6平方公里的文瀛湖成为御东新区的一颗明珠。二是御河蓄水绿化景观工程。今年完成北段1.7公里、南段1公里防渗蓄水工程，东西两岸3.29平方公里绿化景观工程，让这条镶嵌在古城新区之间的绿色长龙，成为大同绿色崛起的标志。三是采凉山森林公园。万亩采凉山、马铺山位于御东新区北部，是新区背山面水的风水宝地和绿色屏障。要加快公墓搬迁，强化环境整治，封山育林，建亭造景，建设规模壮观的森林公园，为福城宝地注入新的生机。四是东城墙环城公园。45万平方米的环城公园，集护城河、下沉式广场、绿化景观、人文雕塑于一体，与气势恢宏的东城墙相映成辉，将成为市民休闲和游客观光的新景区。五是北魏明堂公园。巍峨壮观的北魏明堂主题公园，占地150亩，成荫大树点缀其间，将成为生态名邑新景观。六是儿童公园改造工程。将原320亩公园扩展到540亩，拆除周边侵占公园的不协调建筑，打造功能完善、景观优美的新公园，将绿色公共空间还归人民。七是建设动物园和儿童游乐场。优化大同公园、儿童公园的空间布局，实现城市公园合理分工，让人民群众充分享受绿色环境休闲之美。八是十里河森林公园。去年完成了智家堡垃圾场治理、建设450亩森林公园，今年继续治理十里河粉煤灰场，建设千亩森林公园，让灰尘笼罩的污染世界变成绿色家园。九是口泉植物园。将原来属于矿区、同煤林场的千亩苗圃，改造成为具有现代景观、体现大同植物多样性的植物园，大幅度提升口泉区的生态品质。十是两河湿地公园。在御河、十里河南部交汇处，建设千亩自然生态大景观。十一是梓家村森林公园。将堆积三十余年数百万吨垃圾科学覆盖，广植大树，创造景观，让千亩垃圾山变成绿洲，彻底改善御东新区北部上风上水的生态环境。十二是绿化工程。抓好绕城高速西环绿化、云冈峪绿化、城市道路绿化等工程，让森林走进城市，让城市拥抱森林。

加强环境保护基础工程，建立生态名邑保障体系。新建御东污水处理厂；积极推进十里河生态走廊建设工程，建设和完善8座污水处理厂。加大市区污水管线建设力度，实现雨污分流。实施电厂煤场密闭化改造，推进粉煤灰综合利用，减少煤尘污染；完成集中供热管网延伸121公里，新增集中供热面积800万平方米。完成市区2个垃圾中转站建设，引入封闭式垃圾自动收集系统，提高城市垃圾收集、清运水平，确保垃圾发电厂正常运行。严格落实节能减排目标责任制，注重公共机构节能减排的示范效应，发挥环境保护在促进发展方式转变中的重要作用。

（五）突出项目带动，打造特色农业新亮点

大同农业是弱质产业，生产条件较差，产业化水平低，农民生活艰苦，乡村面貌落后。加大对农业、农村的投入，实施项目带动战略，将中央、省、市、县的惠民政策，真正落实到村、到户，让人民群众得到更多的实惠，是大同农业、农村发展的基本思路。要向上争取更多的项目和资金，集中财力，捆绑使用，切实提高农业项目的投入产出效益。

严格落实强农惠农政策，大幅增加农业农村投入。不折不扣地落实好粮食直补、良种补贴、农业生产资料综合补贴、农机购置补贴等政策，及时给农民兑现。大幅度增加财政对“三农”的投入，逐步提高煤炭可持续发展基金用于“三农”的比例。积极鼓励工商企业特别是资源型企业反哺农业，推动农业产业化水平提升。

加强农业基础设施建设，提高农业综合生产能力。实施好国家京津风沙源治理、首都水资源保护、退耕还林、太行山绿化及省重点造林绿化工程，完成生态建设任务30万亩。完成孤山水库应急水源工程建设。完成册田水库、恒山水库、赵家窑水库和十里河水库除险加固工程。开工建设守口堡、唐河水库。新增节水灌溉面积7.5万亩。解决23万人饮水安全问题。

提高产业化水平，大力发展现代农业。建成雨润集团投资2亿元的百万头生猪屠宰项目，继续培育壮大华晟果蔬等10户领军龙头企业,力争龙头企业达到135户，产值21.5亿元以上。新发展农民专业合作社300个，培训200个农民合作社带头人。推进现代农业示范区建设，建设全省一流的农牧一体化功能区。力争建设温室大棚1.5万栋，新建50个标准化健康养殖小区，培育精品工程20个。完善农产品质量安全体系建设。抓好动植物疫病、质量检测体系建设，完成10个有机、绿色和无公害食品认证。

继续推进新农村建设。完成省确定的重点推进村“四化四改”和社会事业发展“五个一”工程建设任务；进一步巩固85个试点村和467个重点推进村的新农村建设成果。启动100个新农村“环境整治工程”，扶持培育50个样板型新农村建设典型。继续抓好扶贫开发工作。全面完成农村“五个全覆盖”任务。

（六）突出民生改善，打造和谐大同新境界

我们的政府是人民的政府，改善民生是经济发展、社会进步的根本目的。只有着力保障和改善民生，让人民生活得更加幸福、更有尊严，经济发展才有持久的动力，社会进步才有牢固的基础，才能真正实现社会的公正和谐。政府要“重民生、勤民事、薄民赋、保民富、宽民力、从民便、悯民穷、恤民灾、除民害、询民瘼”，把改善和保障民生作为基本工作方向，以民生为纲，把民生作为一切工作的出发点和落脚点。

安居为民生之首。安置房建设是大同当前最重大、最紧迫的民生工程。要坚持把民生工程办成民心、民富、民有、民和、民乐工程，把“民”字放在首位，集中力量，加快建设进度。今年在去年投资50亿元的基础上，再投资50亿元，新开工建设230.4万平方米、27765套安置房，争取上半年竣工10050套，下半年竣工32575套，确保实现当年拆迁当年安置。要坚决保证安置住房的工程质量，真正实现基础设施完善，城市功能配套，生态环境良好，居住品质提升的目标，让人民群众拥有长久保值增值的不动产，做到“拆迁谁、改变谁、造福谁”，得到更多的实惠。

就业为民生之本。坚持把促进就业放在经济社会发展的优先位置，全方位促进就业增长，确保就业形势基本稳定。充分发挥政府投资和重大项目建设的带动作用，城镇新增就业5万人。实施创业、就业工程，带动就业1.23万人。认真做好高校毕业生、农民工、下岗失业人员就业再就业工作，帮助零就业家庭人员就业。

保障为民生之安。继续扩大社会保险覆盖范围，加强基金征缴，重点做好农民工、非公经济组织就业人员、城镇灵活就业人员参加社会保险。进一步完善养老保险关系转移接续办法，积极推进事业单位养老保险改革，探索解决未参保集体企业退休人员养老保险问题，认真做好被征地农民的社会保障工作，争取扩大新型农保试点范围。继续完善城镇基本医疗保险制度和生育保险制度，研究解决破产企业职工医疗保障问题，积极发挥失业保险保生活、促就业、防失业三位一体的功能。加强城乡低保工作，完善社会救助体系。

教育为民生之要。优化教育布局，扩大教育规模，提高教育质量，实现人口增长与教育需求的平衡，让每一个应当接受教育的孩子平等享受良好的教育。优先保证拆迁安置小区的教育配套，全面落实义务教育经费保障制度，改善教育基础条件，大力度推进中小学校舍安全工程。积极筹建大同师范专科学校，推进浑源师范、大同幼师与大同师范资源整合，推进城区八校、三十校与北岳小学合并建校；实施五中、九中、十二中、实验小学迁建工程；新建十六中，改扩建北岳中学。扶持职业教育健康发展，开工建设大同市高级技工学校。

医疗为民生之愿。推进城乡基层医疗卫生机构建设，规划御东新区社区卫生服务机构设置，确保社区卫生服务机构全覆盖。实现乡镇卫生院100%达标，进一步完善支持村卫生室建设的政策措施。推进落实基本药物制度。继续扩大基本医疗保障覆盖面，推进医疗卫生基础设施建设，完成一医院、五医院、六医院、中医院建设工程，为方便人民群众治病创造更多优质资源。加大对重大疾病的监测和防控力度，做好突发公共卫生事件和重大传染病防治工作，启动市疾病预防控制中心、中心血站、检验检测中心综合楼建设工程。认真贯彻《全民健身条例》，促进全民健身活动深入开展。做好人口和计划生育工作，继续稳定低生育水平，提高出生人口质量。

安全为民生之重。以高度负责的态度，强化安全生产意识，杜绝重特大事故发生。牢固确立“抓经济发展是政绩，抓安全生产也是政绩”的理念，强化安全生产和安全监督的主体责任，实现安全生产形势持续稳定。加强社会治安综合治理，建立健全打防管控的长效机制，确保社会治安稳定。积极主动化解社会矛盾和纠纷，畅通信访渠道，引导群众理性合法表达个人诉求。

（七）突出人才战略，打造人民满意的好政府

一个人民满意的服务型政府，必然是有政治理想、有办事能力、有道德操守的政府。建设一支具有较高政治理论素养、专业知识水平、责任担当精神的公务员队伍，是实现大同振兴的当务之急。人才资源是第一资源，能力建设是第一建设。大同需要人才，需要发现和培养各方面优秀人才。要坚持“人才优先，服务发展；以用为本，创新机制；高端引领，实用为主”的指导思想，实施人才优先发展战略，做到人才资源优先开发、人才结构优先调整、人才投入优先保障、人才资本优先积累、人才发展优先规划、人才优秀优先使用，让大同人才辈出、人才当家、人才集聚。

要拓宽人才选拔的视野和渠道，加强同清华、北大、人大等高等院校战略合作，公开择优选择各类专业人才，强化政府管理人才队伍建设。要优化人才使用的环境和氛围，让能干事的有职位，想干事的有机会，干成事的有地位，形成能者上、庸者下的人才倒逼机制。靠能力筛选人，靠业绩使用人，靠制度约束人。要倡导学习的风气，确立正确的价值导向，以读书为荣，以不学为耻；以内行为荣，以外行为耻；以能干为荣，以平庸为耻；以勤奋为荣，以懒惰为耻，建设学习型政府，形成不断学习进步的社会激励机制。要唯才是举，量才使用，讲文凭更讲水平，讲职称更讲称职，讲阅历更讲能力，真正把优秀人才放到重要的岗位，做到以事业留人，以待遇留人，以情感留人。实践是检验干部能力的唯一标准，有能力的干部，坐下来能研究问题、提出思路、拿出办法；走出去能协调工作、解决矛盾、办好事情。懂全局、察民意、善决策、抓重点、敢负责。

抓落实是能力的具体体现。建设服务型政府，必须练好抓落实的基本功，养成“马上办、办得好”的工作思维，做到事不过夜，事无巨细，事必躬亲。强化政务大厅的功能，落实窗口服务，简化办事程序，提高办事效能。继续推进政事分开、事企分开，增强事业单位发展活力。提倡现场工作法，深入一线，靠前指挥，问题在一线发现，矛盾在一线解决，业绩在一线创造，成效在一线检验。一个地方、一个人，用落实代替说话，则兴、则吉、则荣；用说话代替落实，则衰、则凶、则危。

一个有作为的好政府，既要干事，又要干净。清廉操守既是干大事的必要条件，也是衡量能力的重要标尺。有能力、有操守，才是真正有能力的干部。要加强公务员队伍的道德操守修养，常怀敬畏之心，常思贪欲之害，常修为政之德。要加强监督检查，充分发挥监察、审计部门的作用，坚决查处违法违纪大案要案。要创造条件让人民批评政府、监督政府，同时充分发挥新闻舆论、网络媒体的监督作用，让权力在阳光下运行。要自觉接受人大、政协的监督，严格依法行政，努力提高政府的执行力和公信力。

各位代表！大同正走在一个重要的历史关头，勇往直前，柳暗花明，胜利在望；畏难却步，山重水复，功亏一篑。“行百里者半九十”。振兴大同的崇高事业等不得、慢不行、输不起。让我们紧密团结在以胡锦涛同志为总书记的党中央周围，深入贯彻落实科学发展观，在市委的领导下，以浴火重生、蹈海弄潮的豪迈气概，以壮士断腕、破釜沉舟的坚强决心，以只争朝夕、快马加鞭的拼搏精神，以勤奋刻苦、埋头苦干的扎实作风，以不计毁誉、不问收获的博大胸怀，锐意进取，奋发有为，为实现大同“三名一强”的宏伟目标而努力奋斗！
